# Differential use of emotion regulation strategies when engaging in artistic creative activities amongst those with and without depression

**DOI:** 10.1038/s41598-019-46138-3

**Published:** 2019-07-09

**Authors:** Daisy Fancourt, Hannah Ali

**Affiliations:** 1 Department of Behavioural Science and Health, University College London, London, UK; 2 Camden and Islington NHS Foundation Trust, London, UK

**Keywords:** Psychology, Emotion

## Abstract

The ability to effectively regulate our emotions has been shown to be impaired in people with depression. Arts activities have been found to improve depression, but whether people with depression make differential use of emotion regulation strategies (ERSs) when engaging in the arts remains unclear. This study analysed data from 11,248 individuals with depression who were matched on demographics, personality and arts experience with a further 11,248 individuals without depression. We found a significantly lower overall use of self-reported ERSs when engaging in arts amongst those with depression; specifically lower use of approach strategies (e.g. reappraisal) and self-development strategies (e.g. improved self-esteem), but the same use of avoidance strategies (e.g. distraction). However, these differences were very slight (very small effect size and <1% difference). This suggests that people with depression still experience benefits for emotion regulation, which could help to explain the beneficial effects of arts interventions reducing symptoms of depression.

## Introduction

There is a broad literature on the benefits of the arts for mental health. Activities such as singing, drumming, dancing and art-making have been found to reduce mild-moderate depression in populations including children^1^, adults^2,3^, and older adults^4^, and also help to reduce severe depression^5,6^. However, whilst research has shown clear benefits of the arts for depression, what remains unclear is whether people with depression have different emotional responses to arts engagement than people without depression.

Emotion regulation refers to the ability to manage one’s emotional experiences in a way that enables adaptive engagement in daily life within one’s environment^7^. Emotion regulation research grew out of research on coping^8^, but focuses on more transient states (e.g. regulating emotional responses to feeling angry or upset compared to coping with chronic stress or bereavement)^9^. Emotion regulation can be unconscious (employed so early in the sequence of an emotional response that it prevents an ‘unregulated’ emotion from occurring), or it can be part of an explicit strategic process, distinguished from emotional reactivity in having an overall emotion regulation goal^10^.This ability to effectively regulate our emotions is fundamentally linked to our mental health^11–13^, and has been reported to be impaired in some people with mental illness. For example, amongst people with depression, studies have shown individual difficulties in being able to identify one’s own emotions and effectively modulate these emotions^12^. These difficulties appear to be driven by a combination of cognitive mechanisms (e.g. an impaired ability to process negative information)^14^, neurological mechanisms (e.g. hyperactivity in limbic regions during anticipation of or exposure to negative stimuli and dampened prefrontal activity)^12,15^ and biological mechanisms (e.g. over-activation of the hypothalamic-pituitary-adrenal axis and consequent high cortisol production rates)^12^. Other studies have identified specific types of emotion regulation strategies (ERSs) that appear to be used differently in those with and without depression, with three core findings. First, people with depression make less use of strategies that involve either problem solving or reappraisal^14,16^. Both of these types of strategies reduce negative affect and physiological arousal, so are generally considered to be ‘adaptive’ strategies^16,17^. Second, people with depression make more use of strategies focused on avoiding negative emotions (such as suppression)^14,16^. When this suppression relates to negative emotions, it can be ineffective as these negative emotions remain unaddressed, leading to increased physiological arousal and cognitive load^16,18^. When this suppression relates to positive emotions it can increase the severity of depressive symptoms^19^. While certain types of avoidance such as distraction can sometimes be adaptive (such as providing a psychological and physiological break from experiencing negative emotions), distraction has been reported to be used less amongst people with depression^20^. Third, people with depression make greater use of rumination^14^, which, in many studies, has been highlighted as an unhealthy or maladaptive ERS^13,21^.

However, although arts activities have been found to improve depression, whether people with depression make less use of emotion regulation strategies (ERSs) when engaging in the arts remains unclear.

We might hypothesise, in line with the broader research on ERSs in depression, that people with depression might have blunted use of ERSs when engaging in the arts. Yet other studies have suggested that the arts are in fact a particularly effective way of achieving emotion regulation. For example, several studies have demonstrated the beneficial effects of singing and music for regulating emotions including through the adaptive use of avoidance and distraction, reappraisal and even rumination^22^. Similar benefits for emotion regulation have been found from art-making and viewing^23^, dance and movement^24^ and reading^25^. Furthermore, research suggests that interventions that activate positive emotions can break cycles of stress and negative affect^26^. This activation of positive emotions is often bi-directionally related to improvements in cognitive flexibility, creativity, interpersonal behaviours, generosity and cooperation^27,28^: all factors that the arts have been shown to affect. So it is also plausible that the arts could continue to be sources of positive affect and adaptive emotion regulation, even amongst people with depression.

Therefore, this study used a large sample to explore whether individuals with depression make different use of ERSs when engaging in artistic creative activities compared to non-depressed individuals. ‘Artistic creative activities’ were defined in the dataset following a theorised model for population-level research^29^, consisting of performing arts (singing, dancing, playing a musical instrument, rehearsing or performing in a play/drama/opera, learning or practising magic tricks or circus skills), visual arts, design and craft (painting, drawing, printmaking, sculpture, pottery, calligraphy or jewellery making, textile crafts e.g. embroidery, crocheting or knitting, wood crafts such as carving or furniture making), literature-related activities (reading a novel, stories, poetry or plays for pleasure, creative writing, and composing music), and online, digital and electronic arts (creating artworks or animations on a computer, making films or videos, photography). We followed the theoretical standpoint that artistic creative activities are ‘multimodal’ activities^30^: while each artistic activity might have distinct properties different from other artistic activities, all artistic activities involve consistent underlying processes that are inherent to them being ‘artistic’, such as the use of imagination, cognitive stimulation, experiential pleasure, emotional stimulation and the cultivation of individual skills^31^. According to this theoretical approach, the major distinguishing feature between different artistic creative activities is personal preference. Therefore we asked participants to focus on the creative activity they felt was most effective at regulating their emotions for this study and compared responses to all types of ‘artistic creative activities’ together in our analyses. We then tested the consistency of results when focusing on more specific types of artistic creative activities as a secondary research question.

## Results

### Participant characteristics before and after matching

**Figure 1:**
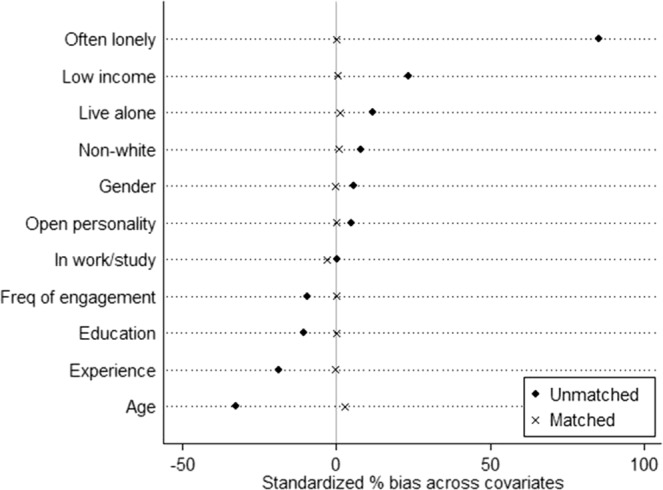
Standardised bias (%) across covariates in the propensity score before and after matching.

Data were collected for this study as part of *The Great British Creativity Test*: a large Citizen Science study in the UK involving 47,924 adults (age 18+). We included data from adults who engage in artistic creative activities at least once a year. 38.1% of these individuals showed symptoms of depression above the threshold of 3+ on the short Centre for Epidemiological Studies Depression (CES-D) scale: a common validated measure of depressive symptoms used in population studies^32^. Participants with depression were on average younger, more likely to be female, had a slightly lower educational attainment, were more likely to earn less than £30,000, more likely to live alone and more likely to often feel lonely (Table 1). On average, they had slightly less experience of doing creative activities and engaged slightly less frequently. We used propensity score matching to match each person with depression with somebody without depression who had similar scores on variables we identified as being potential confounders, such that the only key factor that differed between them was their mental health^33^. These confounders included demographic variables (age, gender, ethnicity, educational attainment, low income and employment status), social variables (whether an individual lived alone and perceived loneliness), creative experience (number of years engaging in artistic creative activities and frequency of engagement), and personality type. This provided matched 11,248 pairs for our analyses (total N = 22,496). Following matching, groups were well balanced (Fig. 1). There was a slight difference in age and employment status between those who were depressed and non-depressed but this only equated to a 4.8 month average age difference and 0.9% more people in the non-depressed sample being in work or study, so these differences were deemed negligible. The success of the overall matching was confirmed using a range of post-estimation tests discussed further in the methods section^34^.

**Table 1:** Demographic characteristics of participants in virtual and live choirs before and after propensity score matching.

	Before matching			After matching		
	Depressed (n = 17,797)	Non-depressed (n = 28,892)	P	Depressed (n = 11,248)	Non-depressed (n = 11,248)	P
Age (mean years, SD)	44.3 (14.3)	49.3 (14.3)	<**0**.**001**	45.4 (13.7)	45.0 (14.1)	**0**.**001**
Gender (female, %)	58.5%	56.2%	<**0**.**001**	57.3%	57.5%	0.82
Ethnicity (white, %)	11.0%	8.4%	<**0**.**001**	9.1%	8.8%	0.47
Educational attainment (%)			<**0**.**001**			0.73
Education to age 16	10.3%	9.2%		9.5%	9.5%	
Education to age 18	18.5%	14.4%		16.3%	15.7%	
Degree	44.3%	46.4%		45.5%	46.8%	
Postgraduate degree	26.9%	30.0%		28.7%	28.0%	
Income below £30,000 (%)	39.5%	28.6%	<**0**.**001**	35.7%	35.4%	0.56
In work/study (%)	74.5%	74.3%	0.73	79.2%	80.3%	**0**.**02**
Living alone (%)	19.7%	15.4%	<**0**.**001**	18.1%	17.6%	0.3
Often feel lonely (%)	32.5%	2.5%	<**0**.**001**	5.6%	5.6%	>0.99
Experience in doing creative activities (%)			<**0**.**001**			0.94
<1 year	3.6%	2.4%		3.4%	2.8%	
1–5 years	12.9%	10.6%		12.1%	12.1%	
6–10 years	11.1%	9.5%		10.4%	10.9%	
10–19 years	19.5%	16.2%		18.6%	19.4%	
20–39 years	31.4%	31.2%		33.0%	32.5%	
40+ years	21.5%	30.2%		22.5%	22.3%	
Frequency of doing creative activities (%)			<**0**.**001**			0.44
Less than once a month	11.0%	8.3%		10.5%	10.7%	
Once or twice a month	14.2%	12.6%		14.9%	13.8%	
Once a week or more	33.8%	36.1%		34.4%	36.0%	
Every day	41.0%	42.9%		40.3%	39.6%	
Open personality [range 3–21] (mean, SD)	13.5 (4.4)	13.3 (4.4)	<**0**.**001**	13.44 (4.3)	13.44 (4.4)	0.99

### Use of ERSs

Emotion regulation strategies were measured using a validated measure specific to artistic creative activities: the Emotion Regulation Strategies for Artistic Creative Activities scale (ERS-ACA). This scale includes a general factor on overall use of ERSs as well as subscales specifically focusing on avoidance strategies (e.g. distraction, suppression or detachment), approach strategies (such as acceptance, reappraisal and problem solving), and self-development strategies (such as enhanced self-identity, improved self-esteem and increased agency). All were scored from 1–5 with higher scores indicating greater use of the strategy^35^.

**Figure 2:**
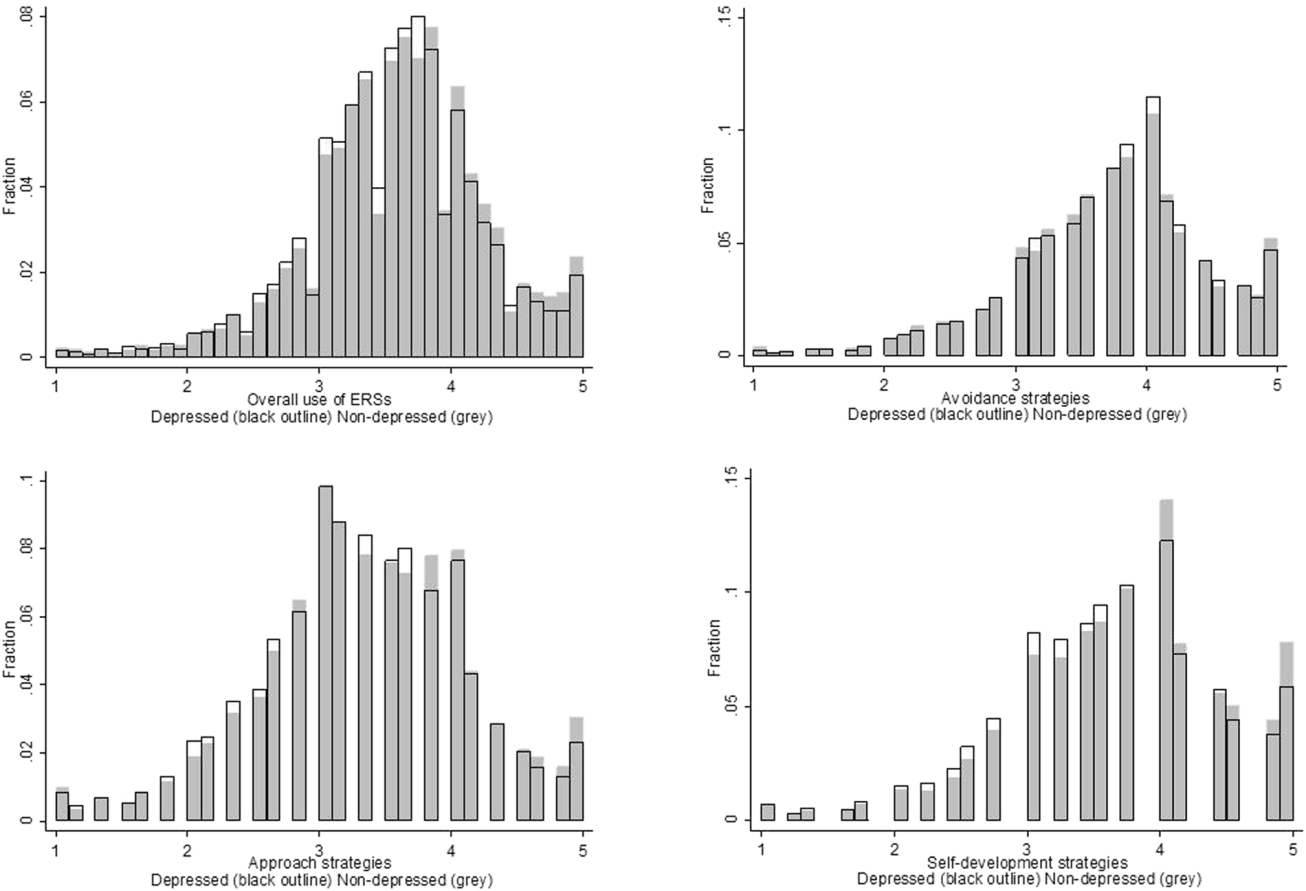
Histograms of scorings for use of ERSs amongst those with and without depression.

Paired analyses of the matched data revealed that there was a slight but significant difference in the overall use of ERSs amongst those with and without depression. Those with depression made marginally less use of strategies overall (mean depressed = 3.56 SD = 0.65, mean non-depressed = 3.59 SD = 0.67, p < 0.001). This was a difference of just 0.8% and a very small effect size, d = 0.032. When considering the subscales, there was no overall difference in use of avoidance strategies amongst those with and without depression (mean depressed = 3.73 SD = 0.73, mean non-depressed = 3.72 SD = 0.75, p = 0.27, difference 0.3%, very small effect size d = 0.01), but there was a slightly lower use of approach strategies (mean depressed = 3.30 SD = 0.79, mean non-depressed = 3.34 SD = 0.80, p < 0.001, difference 0.3%, very small effect size d = 0.036) and self-development strategies (mean depressed = 3.63 SD = 0.81, mean non-depressed = 3.72 SD = 0.80, p < 0.001, difference 2.4%, very small effect size d = 0.080). However, as illustrated in Fig. 2, these differences were only very slight, equating to less than a 1% lower average score on the ERS-ACA scale.

### Sensitivity analyses

We ran a series of sensitivity analyses. In order to test our assumption of the distinction between depressed and non-depressed cases, we ran analyses in two alternative ways. Firstly, we applied a higher cut-off of 4+ on CES-D, and secondly we omitted all participants who had a score of 2 or 3 on CES-D (a ‘borderline depressed’ score). These analyses reduced the sample to 8,521 pairs (17,042 participants) and 7,690 pairs (15,380 participants) respectively, with both sensitivity analyses well-matched between groups on all variables (except with slightly higher age again). The results from both sensitivity analyses repeated the pattern of slightly lower use of overall strategies, approach strategies and self-development strategies amongst participants with depression (see Table 2). We also ran a sensitivity analysis to confirm if we had over-corrected our model, matching on all the same factor except loneliness and personality, in case these in fact overlapped too closely with depression. This provided a slightly larger sample of 14,777 pairs (29,554 participants) but produced almost identical results (see Table 3).

**Table 2:** Sensitivity analyses testing definitions of depression.

Using a higher CES-D threshold N = 17,042	Depressed	Non-depressed	p	Cohen’s d
Overall use of ERSs	3.55 (0.65)	3.60 (0.67)	**<0.001**	0.054
Use of avoidance strategies	3.73 (0.73)	3.73 (0.75)	0.63	0.019
Use of approach strategies	3.30 (0.79)	3.35 (0.81)	**<0.001**	0.044
Use of self-development strategies	3.62 (0.81)	3.72 (0.81)	**<0.001**	0.087
**Using more distinct groups (removing those with CES-D = 2) N = 15**,**380**				
Overall use of ERSs	3.56 (0.65)	3.58 (0.70)	**0.012**	0.021
Use of avoidance strategies	3.74 (0.73)	3.71 (0.78)	0.077	0.028
Use of approach strategies	3.30 (0.79)	3.32 (0.82)	**0.032**	0.018
Use of self-development strategies	3.61 (0.80)	3.71 (0.82)	**<0.001**	0.087

**Table 3:** Sensitivity analyses using more minimal matching.

Using more minimal adjustment N = 29,554	Depressed	Non-depressed	p	Cohen’s d
Overall use of ERSs	3.54 (0.66)	3.58 (0.68)	**<0.001**	0.042
Use of avoidance strategies	3.71 (0.74)	3.70 (0.76)	0.31	0.009
Use of approach strategies	3.28 (0.80)	3.33 (0.80)	**<0.001**	0.044
Use of self-development strategies	3.61 (0.82)	3.70 (0.81)	**<0.001**	0.078

We also ran further sensitivity analyses to test whether our results were consistent for different types of creative activities. In order to have adequate power, we re-sampled from the entire data set for each analysis. Matching was successful across all variables (including age) for all five sub-group analyses. We found that literature-related activities showed exactly the same pattern of overall lower use of ERSs and specifically lower use of approach and self-development strategies. Visual arts and performing arts too showed the same pattern, although only self-development strategies were significantly lower statistically. However, there was a slightly different pattern of results for online arts and other creative activities, with both in fact showing higher use of avoidance strategies (see Table 4).

**Table 4:** Sensitivity analyses testing definitions of artistic creative activities.

Types of arts activities	Depressed	Non-depressed	P	Cohen’s d
**Performing arts N** = **6,350**				
Overall use of ERSs	3.57 (0.64)	3.59 (0.66)	0.15	0.022
Use of avoidance strategies	3.75 (0.71)	3.75 (0.73)	0.88	0
Use of approach strategies	3.29 (0.78)	3.30 (0.79)	0.63	0.009
Use of self-development strategies	3.65 (0.79)	3.72 (0.79)	<0.**001**	0.063
**Visual arts**, **design and craft N = 5**,**066**				
Overall use of ERSs	3.67 (0.61)	3.70 (0.66)	0.097	0.033
Use of avoidance strategies	3.82 (0.68)	3.80 (0.74)	0.32	0.020
Use of approach strategies	3.35 (0.75)	3.39 (0.79)	0.066	0.037
Use of self-development strategies	3.83 (0.74)	3.92 (0.76)	<0.**001**	0.085
**Literature-related activities N = 4**,**794**				
Overall use of ERSs	3.56 (0.64)	3.60 (0.69)	**0**.**02**	0.043
Use of avoidance strategies	3.74 (0.75)	3.72 (0.79)	0.56	0.018
Use of approach strategies	3.41 (0.82)	3.47 (0.84)	**0**.**014**	0.051
Use of self-development strategies	3.48 (0.88)	3.59 (0.88)	<0.**001**	0.088
**Online**, **digital and electronic arts N = 1**,**446**				
Overall use of ERSs	3.46 (0.70)	3.41 (0.72)	0.19	0.050
Use of avoidance strategies	3.60 (0.77)	3.47 (0.83)	**0**.**002**	0.115
Use of approach strategies	3.10 (0.81)	3.10 (0.82)	0.99	0
Use of self-development strategies	3.71 (0.80)	3.71 (0.79)	0.85	0
**Other creative activities N = 4**,**766**				
Overall use of ERSs	3.46 (0.66)	3.46 (0.70)	0.94	0
Use of avoidance strategies	3.63 (0.73)	3.59 (0.76)	**0**.**038**	0.038
Use of approach strategies	3.22 (0.77)	3.24 (0.80)	0.42	0.018
Use of self-development strategies	3.52 (0.76)	3.56 (0.77)	0.10	0.037

Notes: Performing arts (singing; dancing; playing a musical instrument; rehearsing or performing in a play/drama/opera; learning or practising magic tricks or circus skills), visual arts, design and craft (painting, drawing, printmaking, sculpture; pottery, calligraphy or jewellery making; textile crafts e.g. embroidery, crocheting or knitting; wood crafts such as carving or furniture making), literature-related activities (reading a novel, stories, poetry or plays for pleasure; creative writing; and composing music), online, digital and electronic arts (creating artworks or animations on a computer; making films or videos; photography), other creative activities (cooking or baking; gardening).

## Discussion

This study explored the potential differences in self-reported use of emotion regulation strategies (ERSs) when engaging in artistic creative activities amongst those with and without depression. Our findings showed a significant but very small lower use of ERSs amongst those with depression, with specific differences found for use of approach strategies and self-development strategies but no difference in use of avoidance strategies. This pattern was found specifically across performing arts, visual arts, design and craft and literature-related activities but different for online, digital and electronic arts.

On the one hand, the statistical significance of these findings is in line with research suggesting that employment of adaptive emotion regulation strategies is impaired or ineffective amongst those with depression^11–13^. More specifically, the finding of lower use of ‘approach’ strategies aligns with studies showing blunted use of problem solving and reappraisal strategies^14,16^. The finding that avoidance strategies are the only type of ERS not found to be different amongst those with and without depression is supported by research showing the increased use of expressive suppression amongst individuals with depression^14,16^. But it is of note that we found no evidence of higher usage; merely the same level of usage. If we consider distraction as a more specific type of ‘avoidance’ strategy, this finding is supported by some individual studies suggesting that people with depression can use distraction as effectively as non-depressed participants^36^.

On the other hand, the magnitude of the differences between the two groups in use of ERSs is very small (less than 1% for overall use of ERSs, with a very small effect size); arguably of little practical difference and potentially only highlighted through our large sample size. As such, although more marked differences in ERSs have been reported when individuals with depression engage in other activities, it is possible that arts activities are particularly effective in supporting emotion regulation in individuals with depression. Indeed, arts activities combine cognitive flexibility, creativity, interpersonal behaviours, generosity and cooperation; all elements previously found to be bidirectionally related to positive affect and therefore discussed as potential targets in interventions to improve emotion regulation and reduce depressive symptoms^12,26^. If we combine this theory with findings from this study and findings from previous literature suggesting that the arts are in fact very effective for emotion regulation^22–24,37^, this could underlie the beneficial effects for depression noted from a growing number of research studies^5,6^.

In support of the differences in ERSs being negligible between depressed and non-depressed individuals, when considering whether responses varied by type of artistic activity, the differences largely disappeared. For our sub-analyses of performing and visual arts, the small differences in use of ERSs were removed for overall differences, and both approach and avoidance strategies. The only difference remaining was for self-regulation strategies, for which a lower level was reported for those with depression. Low self-esteem and confidence amongst people with depression are widely reported^38^, so it is possible there was lower trait self-esteem that led to this imbalance, especially given intervention studies have reported improvements in self-esteem, confidence and agency in people with depression when they engage in both performing and visual arts activities^39^. When focusing specifically on digital activities, the only difference noted was for avoidance strategies, for which a higher use of avoidance strategies was reported. This could be explained by the highly immersive nature of online activities as compared to other activities. Indeed, part of the addictive nature of digital activities, such as internet usage, is explained by its effects on avoidance strategies^40^.

This study has a number of strengths including its large sample size, its use of a validated measure of depression and of ERSs, and its use of propensity score matching to help achieve exchangeability between people with and without depression. However, although we balanced our participants on all identified confounding factors, it is still possible that latent confounders remain that could have caused imbalance between our depressed and non-depressed samples. For example, we did not consider whether the population had received any active treatment for their depression or had comorbid illness. Further, we relied on participant self-report of use of ERSs when engaging in creative activities rather than experimental manipulation of ERSs with pre-post testing or triangulation of data from multiple sources. However, the measure we used is a validated measure and other studies have used similar approaches for measuring ERSs^35^. Further, given that we know from the literature that those with depression are more likely to negatively self-report, and given we found only very small differences between the groups, our results still suggest that there is very little difference in use of ERSs when engaging in arts activities amongst those with and without depression. As already discussed, this was a non-clinical sample so future studies could explore the findings further in participants who meet more specific diagnostic criteria for depression. Additionally, this sample focused specifically on people who do engage in creative activities. However, it is recognised that people with depression can have altered patterns of engagement in activities and higher levels of apathy, anhedonia and boredom^41^. Whether these people who do not engage with creative activities have even more blunted responses to engagement remains unclear. Therefore a future extension of this study could involve experimental studies comparing ERSs amongst those with and without depression. This study also focused on reporting descriptively the differences in ERSs amongst those with and without depression. Whether depression leads to marginally lower responses to creative activities, or whether people who experience marginally lower responses to creative activities are more likely to develop depression remains to be tested. Lastly, this study took the theoretical standpoint that artistic creative activities can be compared together as a group. Whilst our sensitivity analysis tested the consistency of the findings in more specific categories of similar creative activities, future studies may like to explore the effects of individual activities on emotion regulation in further detail. Studies that explored whether findings can be replicated in other cultures would also help to test the definitions used here both for emotion regulation and creative engagement.

Overall, our findings suggest that there is only a slight difference in use of emotion regulation strategies when engaging in artistic creative activities amongst individuals with and without depression. This suggests that practically people with depression still experience benefits from arts activities for emotion regulation. This could help to explain why arts interventions have been found to have beneficial effects in reducing symptoms amongst people with depression. Given the recognised need to identify further interventions that support emotion regulation in people with depression, interventional studies exploring whether arts activities encourage more effective use of ERSs in broader life are recommended.

## Methods

### Participants and design

Data were collected for this study as part of *The Great British Creativity Test*: a large Citizen Science study in the UK involving 47,924 adults (age 18+) who engage in artistic creative activities at least once a year. The study was publicised via the BBC and available through its website. Participants received no payment for taking part. Participation took around 20 minutes. The study received ethical approval from the University College London Research Ethics Committee and was carried out in accordance with the relevant guidelines and regulations. All participants provided informed consent.

**Figure 3:**
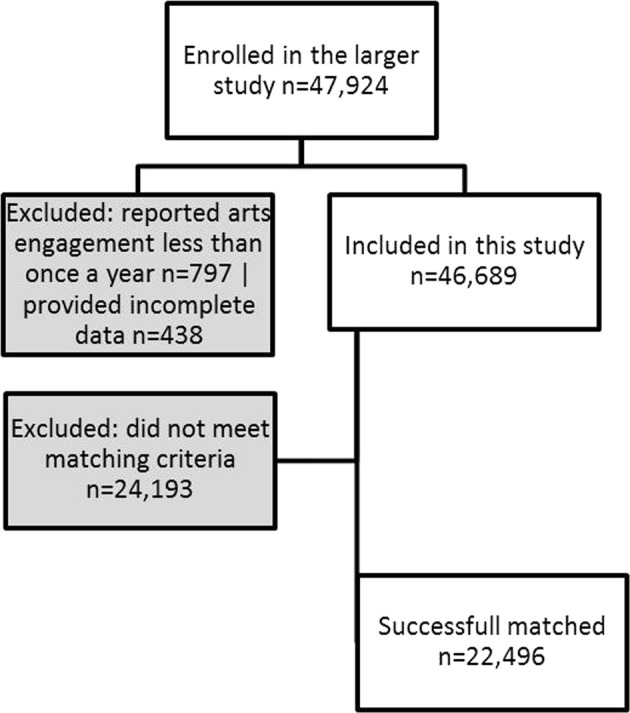
Participant selection for involvement in study matched pair analyses.

Given that having depression is associated with a range of demographic and social factors, and given also that people with depression may have altered behavioural engagement with activities such as creative activities, this study used a propensity matching design. This approach allowed us to match each person with depression with somebody without depression who had similar scores on variables we identified as being potential confounders, such that the only key factor that differed between them was their mental health. The flow of participants into this matched cohort is shown in Fig. 3.

### Measures

Participants provided data for the overall Citizen Science project through online questionnaire. While there are potentially limitless numbers of ERSs, three broad classes have been identified when engaging in artistic creative activities: avoidance strategies (such as distraction, suppression or detachment from negative or stressful emotions), approach strategies (such as acceptance, reappraisal and problem solving), and self-development strategies (such as enhanced self-identity, improved self-esteem and increased agency) (Fancourt, Garnett, Spiro, West, & Mullensiefen, 2019). To measure these emotion regulation strategies, we used the Emotion Regulation Strategies for Artistic Creative Activities scale (ERS-ACA) which includes a general factor on overall use of ERSs as well as subscales specifically focusing on avoidance, approach, and self-development strategies, all scored from 1–5 with higher scores indicating greater use of the strategy (Fancourt *et al*., 2019). The overall scale performed in line with its validation, with an overall Cronbach’s alpha of 0.93 for the entire sample, and subscale alphas of 0.90, 0.88 and 0.88.

Depression was measured using the short Centre for Epidemiological Studies Depression (CES-D) scale: a common self-report measure of depressive symptoms used in population studies. Each item assesses negative affect symptoms or somatic complaints experienced in the past week using a binary reporting scale, with the total number of symptoms summed (0–8). The 8-item version has been found to have comparable psychometric properties to the full 20-item scale. Although not a diagnostic tool, a score of 3 or greater on CES-D 8-item has been validated against standardised psychiatric interviews to indicate the presence of depression^42^.

Demographic questions additionally measured age, gender, ethnicity (white vs other), educational attainment (education to age 16, age 18, degree, or postgraduate degree), income (<£16,000, £16,000-£29,999, £30,000-59,000, £60,000-£89,999, £90,000-£119,000, >£120,000), whether participants lived alone, self-rated loneliness (never, sometimes, often), number of years doing creative activities, frequency of engaging in creative activities, and openness to experience as a personality trait (using a version of the Midlife Development Inventory which measures the five major personality traits^43^.

### Statistics

Analyses were carried out using Stata v14 (StataCorp, College Station, TX). We used propensity score matching; a technique that simulates an experimental setting in an observational data set and creates a treatment group and a control group (the ‘counterfactual’) from the sample^22^. As a result, propensity score matching controls more effectively than regression approaches for the effects of observed confounders, strengthening the causal inference of the relationship. To create a matched cohort of participants with and without depression, we calculated the propensity score (logit model) for each individual based on demographic variables (age, gender, ethnicity, educational attainment, low income and employment status), social variables (whether an individual lived alone and perceived loneliness), creative experience (number of years engaging in creative activities and frequency of engagement), and personality type. We then used nearest available Mahalanobis metric 1-to-1 matching method without replacement, using a caliper size of 0.01 using the Stata module PSMATCH2^33^. Success of the propensity score matching was assessed using Rubin’s B < 25 (B = 4.2), Rubin’s R of 0.5–2 (R = 0.99) and a percentage bias of <10% for each covariate (bias = 0–2.9%)^34^.

For unmatched data, differences between groups (participants with and without depression) were analysed using independent t tests and χ2 tests. For matched data, differences between groups were analysed using paired t tests, Wilcoxon signed-ranks test and McNemar’s test. All tests were two-sided. We further calculated the effect size of findings (Cohen’s d).

We ran a series of sensitivity analyses. In order to test our assumption of the distinction between depressed and non-depressed cases, we ran analyses in two alternative ways. First, we used an alternative cut-off of 4 and above on CES-D, which has been proposed for identifying cases of more marked depression. Second, we excluded any participants with a score of 2 or 3, thereby providing two more distinct groups (score of 0–1 and score of 4+). We additionally tested if we had over-matched our sample by re-running the matching but not including either loneliness or personality as these could be considered too closely related to depression to be suitable matching criteria. Finally, we tested our definition of artistic creative activities by carrying out sub-group analyses for different types of arts engagement.

## Data Availability

Data are publicly available from Open Science Framework.
